# Itaconate ameliorates cardiovascular inflammation in a mouse model of Kawasaki disease vasculitis – brief report

**DOI:** 10.3389/fimmu.2026.1748519

**Published:** 2026-03-27

**Authors:** Youngho Lee, Takahiro Namba, Asli E. Atici, Emily Aubuchon, Malcolm E. Lane, Benjamin L. Ross, Magali Noval Rivas, Moshe Arditi

**Affiliations:** 1 Department of Pediatrics, Cedars-Sinai Guerin Children’s, Cedars−Sinai Health Sciences University, Los Angeles, CA, United States; 2 Infectious and Immunologic Diseases Research Center (IIDRC) and Department of Biomedical Sciences, Cedars−Sinai Health Sciences University, Los Angeles, CA, United States; 3 Smidt Heart Institute, Cedars-Sinai Health Science University, Los Angeles, CA, United States

**Keywords:** IL-1β, itaconate, Kawasaki disease, NLRP3, vasculitis

## Abstract

**Background:**

Kawasaki disease (KD), a systemic vasculitis and the leading cause of acquired heart disease in children, stems from an uncontrolled inflammatory response that fails to resolve in up to 20% of IVIG-treated patients. This treatment resistance increases the risk of cardiovascular complications and highlights the need for more targeted therapeutics.

**Methods:**

We used the *Lactobacillus casei* cell wall extract (LCWE) murine model of KD vasculitis to investigate the therapeutic potential of itaconate, an anti-inflammatory metabolite, in the pathogenesis of KD. The expression of aconitate decarboxylase 1 (*Acod1*), which encodes the mitochondrial enzyme that produces itaconate, was assessed in both samples from KD patients and vascular tissues of LCWE-injected mice. LCWE-injected mice were treated with itaconate, and the severity of LCWE-induced KD vasculitis was evaluated.

**Results:**

LCWE injection led to the development of cardiovascular lesions, specifically aortitis, coronary arteritis, and abdominal aorta dilatation. Expression of *ACOD1* was upregulated in KD patients during the acute phase of the disease and in cardiovascular lesions of LCWE-injected mice. Treatment with itaconate significantly reduced the development of LCWE-induced cardiovascular lesions. Mechanistically, exogenous itaconate suppressed NLRP3 inflammasome activation in LCWE-induced cardiovascular lesions and decreased IL-1β secretion.

**Conclusions:**

Itaconate treatment provides cardiovascular protection in an experimental mouse model of KD vasculitis by decreasing NLRP3 inflammasome activation and reducing vascular inflammation. Itaconate may be a promising therapeutic agent for patients with KD.

## Highlights

- Transcripts of aconitate decarboxylase 1 (*ACOD1/IRG1*), which encodes the enzyme converting cis-aconitate into itaconate, are significantly increased in the whole blood of patients with Kawasaki disease (KD), during the acute phase of the disease, when compared to convalescent-treated KD patients.
- Expression of *Acod1*/*Irg1* transcripts is also significantly increased in cardiovascular lesions of mice in a model of KD vasculitis that mimics human KD pathogenesis.
- Single-cell RNA sequencing of cardiovascular lesions from the mouse model indicates that myeloid cells (macrophages, neutrophils, and eosinophils) are the main sources of *Acod1*.
- Supplementing the Acod1/itaconate axis with itaconic acid reduces the severity of murine KD cardiovascular lesions by decreasing NLRP3 activation in tissue-infiltrating macrophages and IL-1β production.

## Introduction

Kawasaki disease (KD) is an acute pediatric vasculitis and the leading cause of acquired heart disease in children worldwide (1, 2). Despite extensive research, the etiology of KD remains unknown, though it is commonly attributed to an exaggerated immunologic response to an unidentified environmental or infectious trigger in genetically susceptible children (3, 4). Most patients respond to intravenous immunoglobulin (IVIG), which significantly decreases the incidence of coronary artery aneurysms (CAA) (5). Yet, approximately 15–20% of KD patients are IVIG-resistant and remain at very high risk for developing CAAs and long-term cardiovascular complications (1, 6). Therefore, more specific alternative therapeutic approaches are needed not only for the acute phase of the disease, but also for the long-term sequelae that may develop in adulthood.

The *Lactobacillus casei* cell wall extract (LCWE)-induced murine model of KD vasculitis closely mimics key pathological features of human KD, including myocarditis, coronary arteritis, aortitis, and luminal myofibroblast proliferation, resulting in coronary artery dilation and aneurysms (7, 8). In addition to CAA development, systemic artery aneurysms are also observed in patients with KD (9–11). Similarly, LCWE injection in mice leads to dilations in peripheral arteries, including the abdominal aorta (12, 13). Previous studies performed on samples from KD patients and the LCWE-induced KD murine model have emphasized the critical role of IL-1 in the development of KD vasculitis (14, 15). Indeed, increased levels of circulating IL-1β are reported in patients during the acute phase of the disease, and a higher expression of IL-1 transcripts is associated with treatment resistance (16–18). The increased levels of pro-inflammatory cytokines co-occur with the infiltration of neutrophils, macrophages, T cells, and plasma cells into the affected coronary artery, contributing to the inflammatory environment and the subsequent destruction of the coronary artery wall (19–23). LCWE-induced KD vasculitis also reproduces KD immune-associated responses, including activation of the NLR family, pyrin domain-containing 3 (NLRP3) inflammasome, the subsequent production of IL-1β, and the infiltration of macrophages into the inflamed heart and abdominal aorta (13, 24, 25). Indeed, blocking the IL-1β pathway, using mice genetically deficient for either *Nlrp3*, *Il1a*, *Il1b*, or the IL-1 receptor, or through antibody-mediated blockade of the IL-1 pathway, prevents the development of LCWE-induced vasculitis and abdominal aorta dilations (7, 13, 24). Consequently, LCWE-induced KD vasculitis is an invaluable preclinical model that replicates many pathological and immunological hallmarks of human KD, allowing for further elucidation of the mechanisms involved in KD pathogenesis and for the evaluation of potential therapeutic approaches (8).

Itaconate, also known as itaconic acid (ITA), is a mitochondrial immunomodulatory metabolite with potent anti-inflammatory properties (26). Activated macrophages produce itaconate through the decarboxylation of cis-aconitate, a process facilitated by the aconitate decarboxylase I enzyme (*Acod1*, also known as Immune-Responsive gene 1 (*Irg1)*). Itaconate serves as an endogenous regulator of immune cell function and inflammatory responses, limiting tissue damage (27). Itaconate suppresses pro-inflammatory cytokine production while promoting anti-inflammatory responses, positioning it as a potential therapeutic agent for inflammatory diseases (28). Itaconate exerts its anti-inflammatory effects through multiple mechanisms, including by suppressing succinate dehydrogenase (SDH) activity, reducing mitochondrial ROS production, and inhibiting the NLRP3 inflammasome by alkylating cysteine residues in key signaling proteins (26, 29, 30).

While myeloid cells accumulate in the inflamed CA vessels, contributing to KD pathogenesis, whether the anti-inflammatory properties of itaconate may be beneficial in the context of KD vasculitis in an *in vivo* setting has not been investigated yet. Therefore, given the central role of dysregulated inflammation, with increased NLRP3 inflammasome activity and increased IL-1β, in KD pathogenesis, and the demonstrated anti-inflammatory properties of itaconate, we hypothesized that itaconate treatment could ameliorate disease severity in LCWE-induced KD vasculitis. Here, we examined whether pharmacologic administration of itaconate mitigates vascular inflammation and coronary arteritis in the LCWE-induced KD vasculitis model and explored its effects on inflammasome activation and macrophage-mediated cytokine responses.

## Materials and methods

### Mice

All experimental procedures involving mice were approved by and conducted in strict adherence to the guidelines established by the Institutional Animal Care and Use Committee (IACUC) of Cedars-Sinai Medical Center.

Mice were housed at 22 °C with a 12-hour light/12-hour dark cycle and fed a standard chow diet. Wild-type (WT) C57BL/6J mice were bred in our colony at Cedars-Sinai Medical Center. Mice were housed under specific pathogen-free conditions and provided with a standard diet and water *ad libitum.* Mice were randomly assigned to the different experimental groups.

### LCWE-induced murine model of KD vasculitis

*Lactobacillus casei* (ATCC 11578) cell wall extract (LCWE) was prepared as previously published (31). We only used five-week-old male mice for experimental purposes, as LCWE-injection induces stronger and more consistent coronary vasculitis lesions and abdominal aorta aneurysms in male mice than in female mice (13, 24). Mice were injected intraperitoneally (i.p.) with 500 μl of LCWE or an equal volume of PBS. At the indicated experiment endpoints, mice were euthanized by exposure to a lethal concentration of isoflurane (33%), immediately followed by cervical dislocation. Heart tissues were harvested and embedded in Tissue-Tek Optimum Cutting Temperature (O.C.T.) compound (Sakura Finetek, catalog #4583). Abdominal aortas were dissected and photographed. The maximal abdominal aorta diameter was determined by measuring 5 different areas of the infra-renal part of the abdominal aorta in Qupath (https://qupath.github.io). Serial cryosections (7 μm) of heart tissues were stained with hematoxylin and eosin (H&E; MilliporeSigma, catalog MHS32). Representative images were chosen based on corresponding inflammatory scores or aorta measurements reflecting the mean of the sample group.

### Histological analysis

7 μm-thick serial cryosections from OCT-embedded heart tissues were stained with hematoxylin and eosin (H&E, Sigma-Aldrich) for histological analysis or used for immunofluorescent staining. The histopathological examination and assessment of heart inflammation were performed from these sections by a senior investigator blinded to the experimental groups. Heart vessel inflammatory score (coronary arteritis, aortic root vasculitis, and myocarditis) was determined as previously described (31). Only heart sections showing the coronary artery (CA) branch separating from the aorta were scored. Briefly, acute inflammation, chronic inflammation, and connective tissue proliferation were each assessed using the following scoring system: 0 = no inflammation, 1 = rare inflammatory cells, 2 = scattered inflammatory cells, 3 = diffuse infiltrate of inflammatory cells, and 4 = dense clusters of inflammatory cells. Fibrosis was determined using the following scoring system: 0 = no medial fibrosis, 1 = medial fibrosis involving less than 10% of the CA circumference, 2 = medial fibrosis involving 11% to 50% of the CA circumference, 3 = medial fibrosis involving 51% to 75% of the CA circumference, and 4 = medial fibrosis involving more than 75% of the CA circumference. All 4 scores were combined to generate a severity score called the “Heart vessel inflammation score”, as previously published (31). Pictures were taken using a Biorevo BZ-9000 or BZ-X710 microscope (Keyence). Representative images were chosen based on corresponding inflammatory scores that were closest to the mean of the sample group.

### Itaconate treatment

Itaconic acid (ITA) was purchased from Thermo Scientific (Cat. No. A15566.30). The compound was dissolved in the vehicle (sterile PBS) for administration. Mice were i.p. injected with ITA (50 mg/kg body weight) or an equal volume of vehicle (control group) daily from day -1 to day 5 of the experiment. Mice were injected i.p. with either PBS or LCWE at day 0 of the experiment, and the severity of LCWE-induced KD was evaluated at day 7 post-LCWE injection.

### Immunofluorescent staining

IF staining was performed on 7 μm thick frozen tissue sections. Tissue sections were fixed in ice-cold acetone for 5 minutes, washed with PBS, and blocked for 1 hour at room temperature with 3% donkey serum (Abcam; ab7475) in PBS. Sections were then incubated with primary antibodies. Caspase-1 activity in tissues was detected using the fluorochrome-labeled inhibitors of caspases assay (FLICA), according to the manufacturer’s protocol. Frozen heart sections were initially incubated with FAM-FLICA caspase-1 detection reagent (Immunochemistry Technologies, Cat. No. NC0585813) diluted 1:100 for 1 hour at room temperature. Following FLICA treatment, sections were fixed with 10% neutral buffered formalin solution (Medical Chemical, Cat. No. 575A-2.5GL) for 5 minutes. Subsequently, sections were permeabilized with 0.1% Triton X-100 in phosphate-buffered saline for 5 minutes. For NLRP3 detection, sections were incubated with anti-NLRP3 monoclonal antibody (Invitrogen, clone 768319) diluted 1:100 or rat IgG2a isotype control antibody (1:100) for 1 hour at room temperature. Following primary antibody incubation, sections were washed and incubated with donkey anti-rat Alexa Fluor 594-conjugated secondary antibody (Invitrogen, Cat. No. A21209) diluted 1:1000 for 1 hour at room temperature, prepared in blocking solution, followed by another washing step with PBS. Images were obtained using a Biorevo BZ-X710 (Keyence) fluorescent microscope and were further analyzed with ImageJ (NIH) software. For all IF staining, isotype controls for each primary antibody were used to distinguish positive staining from background. Quantification of FLICA and NLRP3 double-positive cells was performed in tissues surrounding coronary arteries and the aortic root using the QuPath software. Whole tissue sections were analyzed for each experimental group. In a separate experiment, mouse heart sections were subjected to IF double staining to detect F4/80 and inducible nitric oxide synthase (iNOS) expression. OCT frozen 7 μm heart tissue sections were fixed in acetone, washed in PBS, and stained with F4/80 monoclonal antibody Alexa Fluor 647 (Clone BM8, BioLegend Cat# 123122, dilution 1:100), purified anti-mouse iNOS antibody (Clone 11F6, BD Biosciences Cat# 610330, dilution 1:100). To distinguish target staining from background, control sections were stained with Alexa Fluor^®^ 647 Rat IgG2a isotype control antibody (Clone RTK2758, BioLegend Cat# 400526), mouse IgG2a isotype control antibody (eBioscience Cat# 11-4724-42). Nuclei were counterstained with Mounting Medium with DAPI (Abcam). Images were obtained using a Biorevo BZ-X710 (Keyence) fluorescent microscope and analyzed in the BZ-analyzer software (Keyence). The acquired images were analyzed using QuPath (version 0.5.1, https://qupath.github.io/). Double-positive cells, indicating co-expression of F4/80 and iNOS, were identified and quantified using QuPath’s cell detection feature. Parameters were set to detect cells based on size, shape, and fluorescence intensity thresholds specific to the fluorophores used. The accuracy of cell detection was confirmed by manual inspection of randomly selected fields. Data are presented as the total number of single-positive and double-positive cells in the total lesion area in each section (n=4/group).

### IL-1β quantification in peritoneal lavage

Mice were i.p. injected with ITA (50 mg/kg body weight) or an equal volume of vehicle solution (sterile PBS) daily from day -2 to day 0. On day 0, LCWE was administered i.p., and 24 hours later, mice were euthanized, and the peritoneal lavage was collected, centrifuged at 400*g* at 4 °C for 5 minutes, and the cells were discarded. IL-1β levels were assessed in the supernatants by ELISA (R&D Systems, Mouse IL-1 beta/IL-1F2 DuoSet ELISA; DY401) according to the manufacturer’s protocol.

### Bulk and single-cell RNA sequencing analysis

A bulk RNA sequencing dataset from whole blood of acute KD (n=105) and convalescent KD (n=31) patients (GSE178491 (32)), and a bulk RNA sequencing dataset that we have generated from murine abdominal aorta of PBS and LCWE-injected mice (n=5/group) (GSE141072 (24)) were accessed via Gene Expression Omnibus (GEO) and analyzed for differential expression (|Log_2_(Fold Change (FC))| > 1 & FDR *p-value* < 0.05) via R package DESeq2 (33) and visualized in volcano plots with ggplot2. A single-cell RNA-sequencing dataset (GSE178765 (12)) that we have generated from the abdominal aortas of PBS (9 tissues pooled) and LCWE-injected (7 tissues pooled) mice, as well as a spatial gene expression dataset (10x Visium; GSE178799 (12)) that we have generated from heart tissue sections of a PBS and a LCWE-injected mouse, were accessed via GEO. Cell populations from both LCWE-injected and PBS-injected abdominal aortas were projected via the *RunUMAP()* function in Seurat (34) and visualized using ggplot2, and expression in specific myeloid subsets of interest in the LCWE aortas was visualized with Seurat’s (34) *VlnPlot() function*. Visualization of the spatial expression of *Acod1* in both LCWE-injected and PBS-injected mouse hearts was done via Seurat ‘s (34) *SpatialFeaturePlot()* function.

### Statistical analysis

All graphs and statistical analyses were performed with GraphPad Prism 10 (GraphPad Software, Inc.). For comparisons of 2 groups, a 2-tailed unpaired Student’s t-test, with Welch’s correction when indicated, was used for normally distributed data. For nonparametric data, the Mann-Whitney two-tailed U/Wilcoxon rank test was used. For more than 2 group comparisons, one-way ANOVA with Tukey post-test analysis was used for normally distributed data. The Kruskal-Wallis test with Dunn’s multiple comparisons test was used for non-normally distributed data. A two-way ANOVA was used when there were two independent variables with multiple groups, such as genotype and treatment. Results are reported as means ± SEM, where each point represents one sample.

### Data availability

Data supporting this study’s findings are available from the corresponding authors upon reasonable request.

## Results

### *ACOD1* expression in whole blood of acute KD patients and cardiovascular lesions of LCWE-injected mice

We first examined the expression of *ACOD1* (also known as *IRG1*), which encodes the enzyme responsible for itaconate production, in a publicly available gene expression dataset (GSE178491 (32)) generated from whole blood samples of acute KD patients (n=105) and IVIG-treated convalescent KD patients (n=31). *ACOD1* was significantly increased (Log_2_ Fold Change (FC) = 2.9; FDR *p-value* = 2.02^-07^) in acute KD patients compared to convalescent KD patients (**Supplementary Figure 1A**).

**Figure 1:**
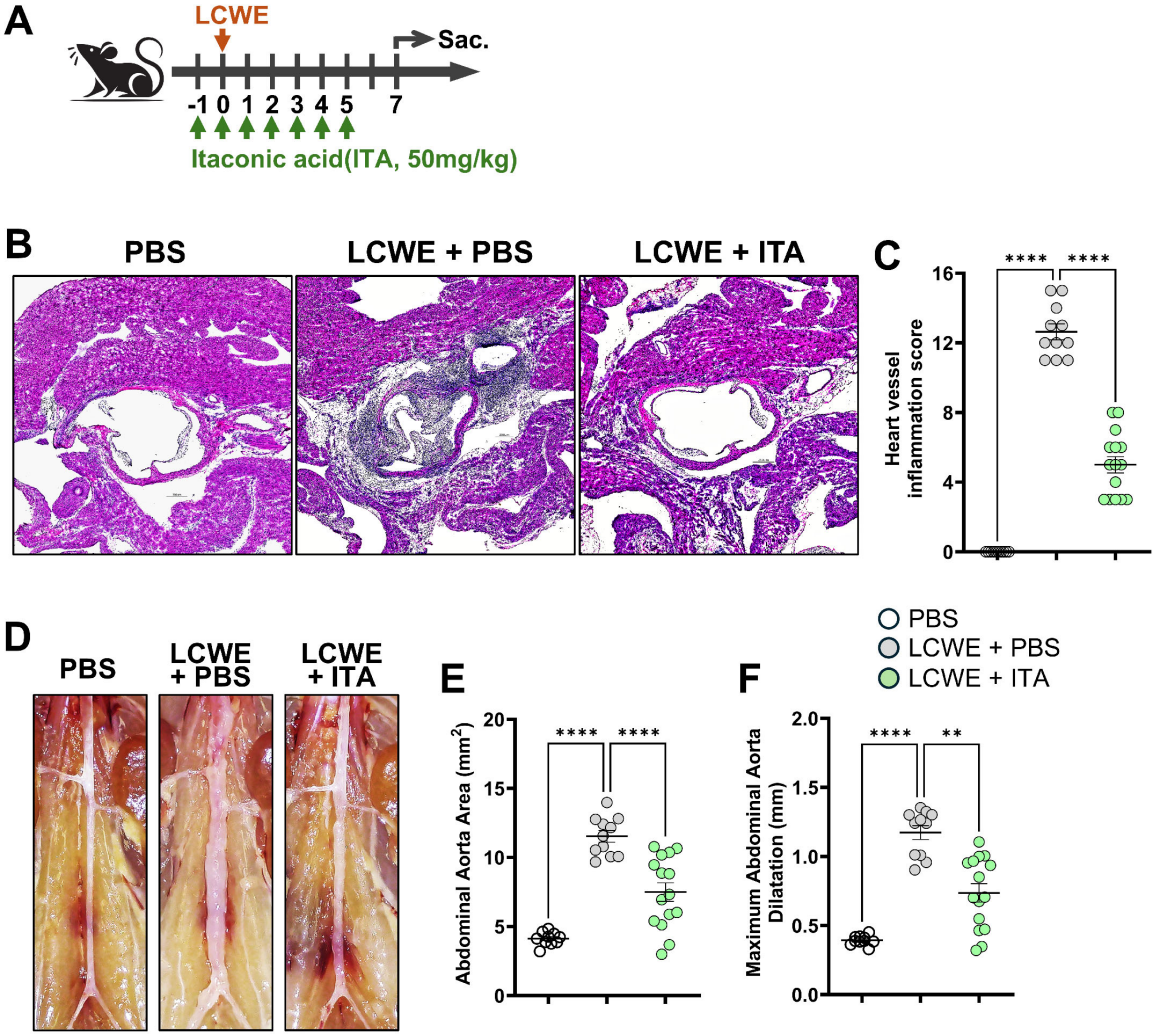
Itaconate given prior to LCWE injection decreases the severity of LCWE-induced cardiovascular lesions. **(A)** Schematic of the experimental design. WT mice were i.p. injected daily for 6 days with either vehicle (PBS) or itaconic acid (ITA), starting one day before either PBS (control mice) or LCWE injection. The severity of LCWE-induced KD was assessed one week post-LCWE injection. **(B, C)** Representative hematoxylin and eosin (H&E)-stained heart sections **(B)** and heart vessel inflammation score **(C)** from PBS or LCWE-injected mice treated or not with ITA at one week post-LCWE injection (n=11 to 15/group). Scale bars, 200 µm. **(D–F)** Representative pictures of the abdominal aorta area **(D)**, abdominal aorta area measurements **(E)** and maximal abdominal aorta diameter **(F)** of PBS-injected and LCWE-injected mice untreated (PBS) or treated with ITA starting the day before PBS or LCWE injection (n=11 to 15/group). Severity of LCWE-induced KD was assessed one week post-LCWE injection. Data presented as the mean ± SEM. Each symbol represents one mouse. **p < 0.01, ****p < 0.0001 obtained by One-Way ANOVA with Tukey’s correction **(C, E)**, Dunn’s correction **(F)**.

Since KD mortality is low (<0.01%) and there is a lack of accessible coronary artery tissues from KD patients, determining which cells express *ACOD1* locally in the coronary artery is challenging. Therefore, we next investigated the expression of *Acod1* in cardiovascular lesions of mice with LCWE-induced KD vasculitis, a well-established preclinical KD model (8, 12, 13, 24, 25, 31). Compared to PBS-injected control WT mice, LCWE injection leads to the development of heart vessel inflammation, specifically coronary arteritis and aortitis, as well as abdominal aorta dilations (**Figure 1**). We first assessed the expression *of Acod1* in our previously published bulk RNA sequencing dataset (GSE141072 (24)) that we generated from the abdominal aorta of PBS-injected control mice (n = 5) and LCWE-injected mice (n = 5). In parallel to the findings in human patients, *Acod1* expression was increased (Log_2_ FC = 7.8; FDR *p-value* = 3.07^-07^) in the abdominal aorta dilations of LCWE-injected mice compared to PBS-injected control mice (**Supplementary Figure 1B**). To identify the relevant cell types, we analyzed the expression of *Acod1 (Irg1)* in a publicly available single-cell RNA sequencing (scRNA-seq) dataset that we also previously generated from the abdominal aortas of PBS (pool of 9 tissues) and LCWE-injected (pool of 7 tissues) mice (GSE178765) (12). *Acod1* was mainly expressed by myeloid cells infiltrating the abdominal aorta dilations, such as monocytes, macrophages, and neutrophils (**Supplementary Figures 2A, B**). We also assessed the expression of *Acod1* in a spatial transcriptomic dataset that we have generated from heart tissues of PBS and LCWE-injected mice, as previously published (12). *Acod1* transcripts were increased in a heart tissue section from an LCWE-injected mouse, and localized specifically around the inflamed coronary artery (**Supplementary Figure 2C**), an area that we previously demonstrated is infiltrated by myeloid cells, including monocytes, macrophages, and dendritic cells (12).

### Itaconate reduces the severity of LCWE-induced cardiovascular lesions

**Figure 2:**
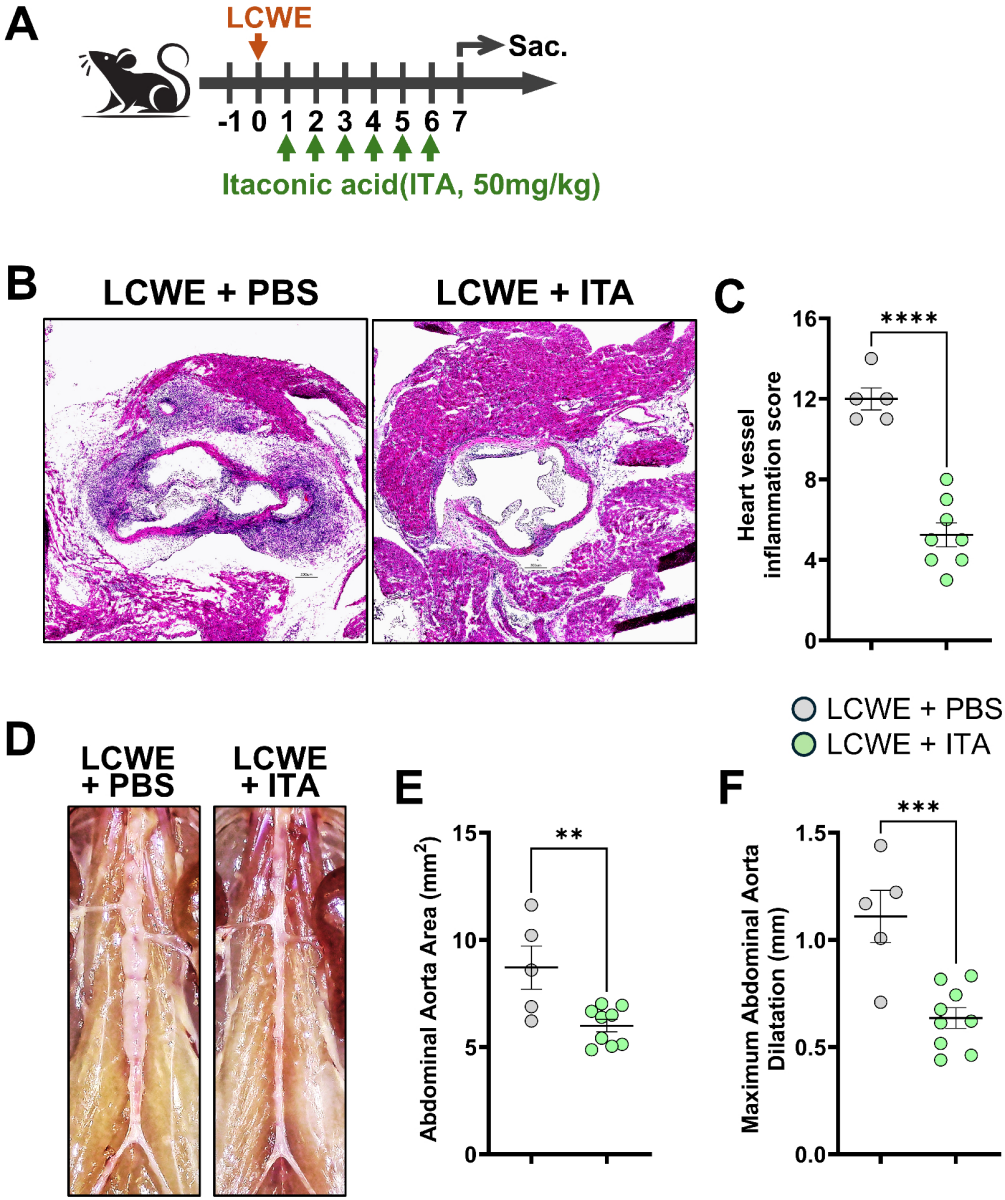
Itaconate given after LCWE injection decreases the severity of LCWE-induced cardiovascular lesions. **(A)** Schematic of the experimental design. WT mice were i.p. injected daily for 6 days with either vehicle (PBS) or itaconic acid (ITA), starting one day after LCWE injection. The severity of LCWE-induced KD was assessed one week post-LCWE injection. **(B, C)** Representative hematoxylin and eosin (H&E)-stained heart sections **(B)** and heart vessel inflammation score **(C)** from LCWE-injected mice untreated (PBS) or treated with ITA at one day after LCWE injection (n=5-9/group). Scale bars, 200 µm. **(D–F)** Representative pictures of the abdominal aorta area **(D)**, abdominal aorta area measurements **(E)** and maximal abdominal aorta diameter **(F)** of LCWE-injected mice treated with PBS or treated with ITA starting from day 1 of LCWE injection (n=5-9/group). Severity of LCWE-induced KD was assessed one week post-LCWE injection. Data presented as the mean ± SEM. Each symbol represents one mouse. **p < 0.01, ***p < 0.001, ****p < 0.0001 obtained by unpaired two-tailed Student’s *t*-test.

To investigate the therapeutic potential of itaconate in KD vasculitis, we treated WT mice with ITA for 5 days starting the day before LCWE injection (**Figure 1A**). Histological examination of cardiac tissues using H&E staining revealed marked differences in vascular inflammatory responses across treatment groups. LCWE injection induced severe cardiac vessel inflammation characterized by inflammatory cell infiltration, vessel wall thickening, and perivascular inflammatory changes (**Figure 1B**). Quantitative analysis of heart vessels demonstrated a significant increase in inflammation severity following LCWE treatment compared to PBS controls (**Figures 1B, C**). Remarkably, concurrent administration of ITA with LCWE attenuated this inflammatory response, with ITA-treated animals showing significantly reduced cardiac vessel inflammation scores, indicating potent anti-inflammatory efficacy of ITA in this murine model of KD vasculitis (**Figures 1B, C**). Evaluation of abdominal aortic dilations and aneurysm formation revealed a similar and significant protective effect of ITA treatment in LCWE-induced KD vasculitis. Compared to control PBS-injected mice, LCWE injection led to the development of aortic dilation and an increase in abdominal aortic area. However, mice treated with ITA exhibited marked protection against these abdominal aortic changes, with significantly reduced aortic dilatation and abdominal aortic area, demonstrating the vascular protective properties of ITA (**Figures 1D–F**). We have also repeated the same experiment after we injected LCWE and induce KD vasculitis to investigate the therapeutic effect of ITA. Mice were first injected with LCWE and ITA treatment was initiated a day after the LCWE injection for 6 days. Mice treated with ITA one day following LCWE injection also showed significantly diminished cardiovascular lesions (**Figure 2**).

### Itaconate attenuates infiltration of NLRP3/FLICA double-positive cells into heart tissues of LCWE-injected mice and reduces IL-1β levels

**Figure 3:**
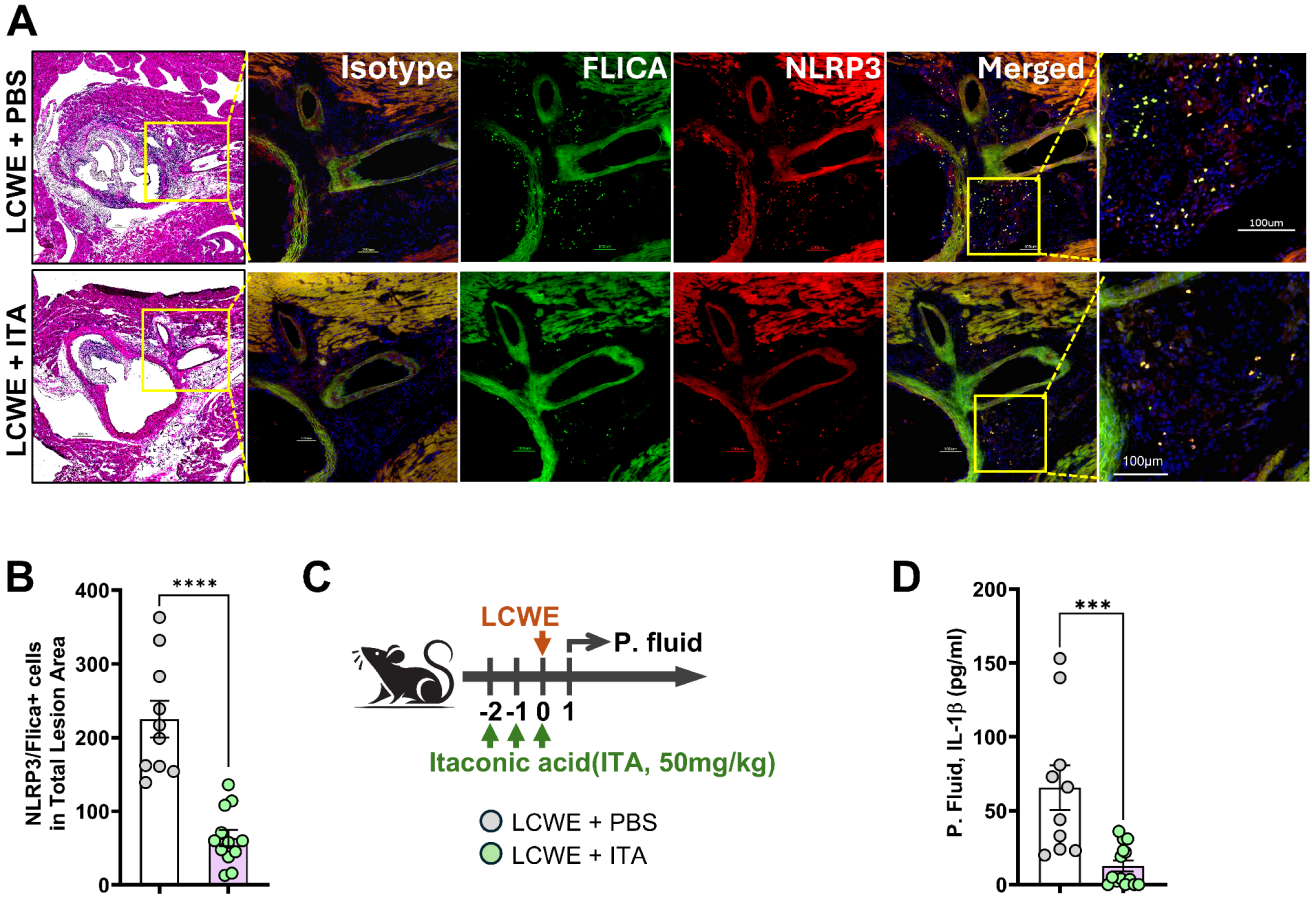
Itaconate attenuates NLRP3/FLICA double-positive cell infiltration in heart tissue and IL-1β secretion in peritoneal lavage of LCWE-injected mice. **(A)** Representative immunofluorescence images showing H&E, FLICA (green), and NLRP3 (red) staining in heart sections from LCWE-injected mice, untreated or treated daily with itaconic acid (ITA) for 6 days starting the day before LCWE injection (n=10 to 12/group). Scale bar is 100μm. **(B)** Quantification of NLRP3/FLICA double-positive cells in heart tissue sections from LCWE-injected mice, untreated or treated daily with ITA for 6 days starting the day before LCWE injection. **(C)** Schematic of the experimental design. WT mice were i.p. injected daily for 2 days with either vehicle or ITA (50 mg/kg), starting two day before injection of LCWE. Peritoneal lavage was collected 24 hours later. **(D)** ELISA measurement of IL-1β levels in peritoneal lavage. Data are presented as mean ± SEM. Each symbol represents one mouse. ***p < 0.001, ****p < 0.0001 by unpaired two-tailed Student’s *t*-test with Welch’s correction **(B)**, Mann-Whitney correction **(D)**.

To elucidate the molecular mechanisms underlying itaconate’s anti-inflammatory effects in this model, we examined inflammasome activation using dual immunofluorescence staining for NLRP3 and FLICA. NLRP3 serves as a key component of the inflammasome complex, while FLICA detects active caspase-1, providing a direct readout of inflammasome activation. Confirming our previous studies (13, 25, 35), LCWE injection dramatically increased the abundance of NLRP3/FLICA double-positive cells throughout cardiac lesions, indicating robust inflammasome activation and supporting that this pathway is a key mediator of LCWE-induced cardiac inflammation. We have previously shown that the majority of these infiltrating NLRP3/FLICA-positive cells are F4/80^+^ cells (12, 13, 25, 35). Treatment with ITA significantly suppressed this NLRP3 inflammasome response, as evidenced by a marked reduction in NLRP3/FLICA double-positive cells within the cardiac lesions (**Figures 3A, B**). To further validate the inhibitory effect of ITA on inflammasome activation *in vivo*, we assessed IL-1β secretion in peritoneal lavage one day after LCWE injection. Mice were treated daily with ITA or an equivalent volume of PBS (control) starting 2 days before LCWE injection, and peritoneal lavage was collected one day after LCWE injection (**Figure 3C**). The levels of IL-1β were significantly increased in the peritoneal lavage of LCWE-injected mice, and this effect was significantly attenuated by pretreatment with ITA (**Figure 3D**). Collectively, these results demonstrate that ITA treatment provides significant protection against the development of cardiovascular lesions in this experimental model of LCWE-induced KD vasculitis by inhibiting the NLRP3 inflammasome-IL-1β pathway. These findings are also consistent with our prior publications, which demonstrate the key involvement of the NLRP3 inflammasome and IL-1β in the development of cardiovascular lesions in this experimental model (12, 13, 25, 35). The therapeutic efficacy of itaconate spans reduction of coronary artery and aortic vessel inflammation, prevention of aortic dilatation and remodeling, and suppression of inflammasome-mediated inflammatory responses, positioning itaconate as a promising therapeutic candidate for IVIG-resistant KD patients who are at higher risk of developing cardiovascular complications.

### Itaconate attenuates F4/80- and inducible nitric oxide synthase-double-positive cell infiltration in heart tissue of LCWE-injected mice

**Figure 4:**
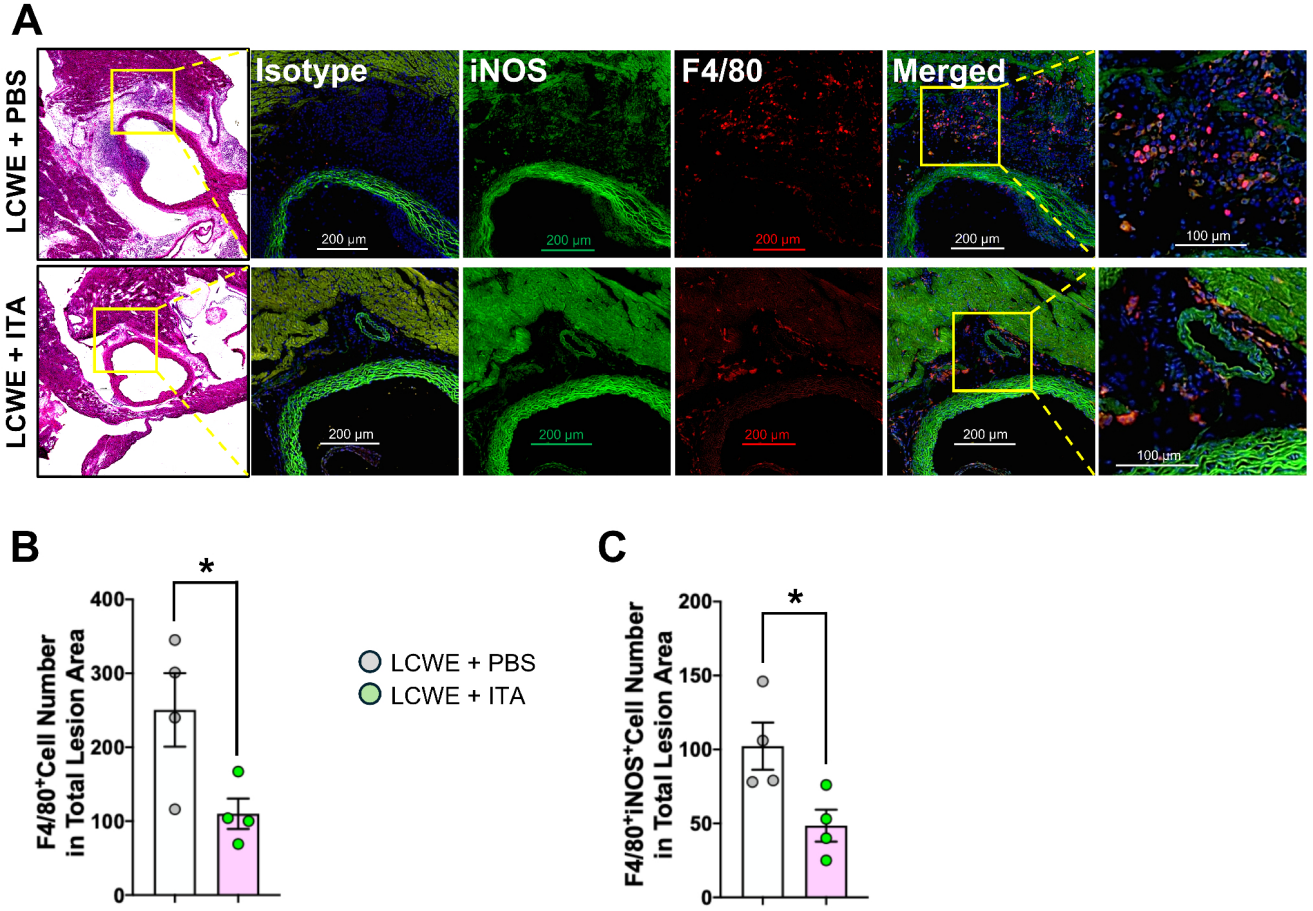
Itaconate attenuates F4/80- and inducible nitric oxide synthase (iNOS)-double-positive cell infiltration in heart tissue of LCWE-injected mice. **(A)** Representative H&E staining and immunofluorescence staining of F4/80(red) and iNOS(green) in serial heart sections of WT mice injected with LCWE, or LCWE-injected mice treated with itaconic acid (ITA) for 6 days starting the day before LCWE injection. **(B, C)** Quantification of F4/80+ cell counts and F4/80+iNOS+ cell counts in the total lesion area of WT mice injected with LCWE, or LCWE-injected mice treated with itaconic acid (ITA) for 6 days starting the day before LCWE injection. Scale bars: 200 μm (H&E) and 100 μm (immunofluorescence). Each symbol represents 1 mouse. Results presented as mean ± SEM. *p < 0.05 obtained by unpaired 2-tailed Student’s t-test.

To further assess the mechanisms underlying itaconate’s anti-inflammatory effects in this model, immunofluorescence staining for F4/80 and inducible nitric oxide synthase (iNOS) was performed to quantify total and classically activated (M1-like) macrophages (**Figure 4**). A significant reduction of the total number of macrophages was observed in the heart lesion area in mice undergoing ITA treatment compared with controls (**Figure 4**). Further characterization of M1-like macrophages showed a significant reduction in iNOS-positive macrophages in the heart lesion area in mice undergoing ITA treatment compared with controls (**Figure 4**).

## Discussion

While the timely administration of IVIG decreases the incidence of CAAs in KD patients, up to 20% of patients are IVIG-resistant and have a 9-fold increased risk of developing cardiovascular complications (1, 6). Since KD is an immune-mediated vasculitis, targeting the inflammatory processes specifically involved in its pathogenesis may reduce the development of cardiovascular lesions and the potential long-term sequelae stemming from coronary artery remodeling more effectively. Both animal models of KD and clinical studies in KD patients indicate that IL-1β is a crucial inflammatory mediator contributing to KD vasculitis (14, 15). Despite several case reports showing promising results using Anakinra, an IL-1 receptor antagonist, to treat IVIG-resistant KD patients (36–39), targeting the IL-1 pathway is not yet the standard of care for treating KD, as it awaits the results of the ongoing phase III clinical trials (40–42). Here, we demonstrate that itaconate markedly attenuates coronary arteritis and abdominal aortic dilation in the LCWE-induced KD vasculitis. We show that treatment with ITA significantly reduces NLRP3 inflammasome activation, as evidenced by a decrease in FLICA-positive (active caspase-1) cells within vascular lesions and a decrease in IL-1β production. These findings identify itaconate as a potent modulator of inflammasome-driven vascular inflammation in the KD vasculitis model. The anti-inflammatory actions of itaconate are consistent with its established role in limiting macrophage activation through the electrophilic modification of NLRP3 and inhibition of downstream caspase-1 activation (26, 29, 30). By suppressing IL-1β–dependent vascular injury, itaconate effectively interrupts a key pathogenic axis implicated in KD vasculitis.

ACOD1, or aconitate decarboxylase 1, is a mitochondrial enzyme that produces the metabolite itaconate from *cis*-aconitate in the tricarboxylic acid (TCA) cycle to regulate inflammation and metabolism (26, 27, 30). Under inflammatory conditions, immune cells, such as macrophages, increase ACOD1 expression and itaconate production (26, 27, 29, 30). Itaconate functions as an anti-inflammatory agent by inhibiting succinate dehydrogenase and specific glycolytic enzymes, protecting against oxidative stress, and reducing the production of type I interferon and IL-1β (43, 44). The upregulation of ACOD1 expression that we observed in both the blood of acute KD patients and the aortic tissues of LCWE-treated mice suggests an endogenous compensatory response that may be insufficient during severe inflammatory states, supporting the therapeutic rationale for exogenous itaconate treatment in IVIG-resistant KD patients.

In a recent study using human coronary artery endothelial cells (HCAECs) as an *in vitro* model referred to as “IVIG-resistant KD”, investigators reported that while high-dose IVIG treatment completely inhibited TNF-α-induced inflammatory responses, IL-1β-induced responses were entirely refractory to IVIG treatment (45). However, they observed that dimethyl itaconate, another membrane-permeable derivative of itaconate, significantly suppressed IL-1β-induced expression of inflammatory markers and cytokines. The authors concluded that dimethyl itaconate or its analog, dimethyl fumarate, which is already in clinical use in oral form for multiple sclerosis and psoriasis (46, 47), may be therapeutically used as a novel drug to alleviate inflammation in severe IVIG-resistant KD patients (45). Our *in vivo* results with the LCWE-induced model of KD vasculitis support these *in vitro* observations.

While mice are widely used to study disease pathogenesis and associated immune responses, some immunological differences exist between humans and mice, which may constitute a limitation to extrapolate murine studies to human disease (48). Among these differences, the ratio of circulating lymphocytes and neutrophils differs, with a high neutrophil predominance in human blood (up to 70%), whereas mice have a higher blood content of lymphocytes and lower frequencies of circulating neutrophils (48). While the functional consequences of these immunological differences remain unclear, these distinctions warrant careful and cautious interpretation to translate experimental findings to human disease. However, fundamental inflammatory and vascular signaling pathways are evolutionarily conserved (49) and murine models uniquely enable controlled mechanistic dissection through pharmacologic and genetic manipulation and temporal disease modeling. Consequently, well-validated mouse systems like the LCWE-induced KD vasculitis model remain indispensable for identifying conserved pathways that drive human inflammatory disease. Our previous work (25, 31, 50) and work by others (51, 52) have demonstrated that the LCWE-induced KD model is a strong preclinical model for studying KD vasculitis, as it recapitulates key pathological features of KD, including coronary arteritis, aortitis, myocardial dysfunction, and immune cell infiltrates in the inflamed area of the CA associated with a strong upregulation of genes and mediators of the NLRP3/IL-1β pathway (8, 14, 53). Indeed, the LCWE-induced murine model of KD vasculitis has demonstrated strong translational relevance, as therapies effective in patients, including IVIG, TNF-α blockade, and IL-1 receptor antagonism with anakinra, have similarly shown efficacy in preventing coronary arteritis and the cardiovascular lesions of KD in this experimental model (25, 31, 50).

Rising rates of IVIG resistance, the high cost and limited global availability of IVIG, and the ongoing risk of coronary artery complications underscore the urgent need to identify novel, accessible, and mechanistically targeted therapies for KD. Given the clinical challenge of increasing IVIG resistance and the growing evidence supporting IL-1–targeted therapies in KD vasculitis, our results suggest that itaconate or its cell-permeable derivatives may represent a novel therapeutic approach to prevent or limit coronary arteritis in KD patients who are unresponsive to standard IVIG therapy. As an endogenously produced metabolite with potent anti-inflammatory properties, itaconate represents a promising therapeutic candidate for KD and other NLRP3 inflammasome-mediated diseases, though comprehensive safety evaluation and clinical trials are needed to optimize dosing and validate efficacy in human patients.

## Data Availability

The raw data supporting the conclusions of this article will be made available by the authors, without undue reservation.
