# Supplementary material for: Itaconate ameliorates cardiovascular inflammation in a mouse model of Kawasaki disease vasculitis – brief report
Source: Front Immunol. 2026 Mar 27;17:1748519. doi: 10.3389/fimmu.2026.1748519 (PMC13065724; doi:10.3389/fimmu.2026.1748519)
Supplement: Supplementary file 1 [file DataSheet1.pdf]

Supplementary Figure 1

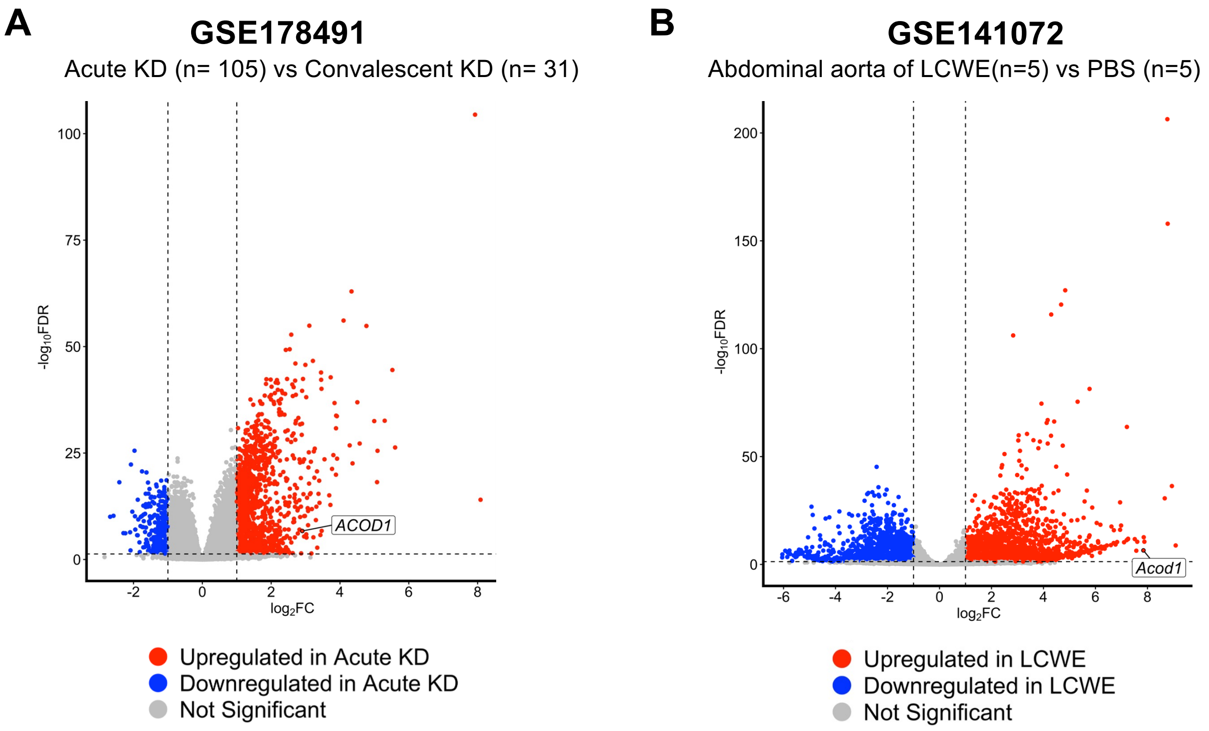

Supplementary Figure 1. Increased *ACOD1* gene transcripts in whole blood of acute KD patients and in the abdominal aorta of LCWE-injected mice.

(A) Volcano plots showing differential expression of *ACOD1* in blood samples from acute (n=105) versus convalescent IVIG-treated KD (n=31) patients (GSE178491). Differential gene expression (DEG) was determined based on a $\log_2$ Fold Change (FC) > 1 and a FDR *p-value* < 0.05. Red color indicates upregulation in acute KD, blue color indicates downregulation in acute KD, and grey color indicates no significant changes in gene expression. (**B**) Volcano plots showing differential expression of *ACOD1* in the abdominal aorta tissues of PBS-injected control mice (n=5) and LCWE-injected mice (n=5) developing abdominal aorta dilations at 2 weeks post LCWE-injection (GSE141072). DEG was determined based on a $\log_2$ Fold Change (FC) > 1 and a FDR *p-value* < 0.05. Red color indicates upregulation in LCWE-injected mice, blue color indicates downregulation in LCWE-injected mice, and grey color indicates no significant changes in *Acod 1* gene expression.

Supplementary Figure 2

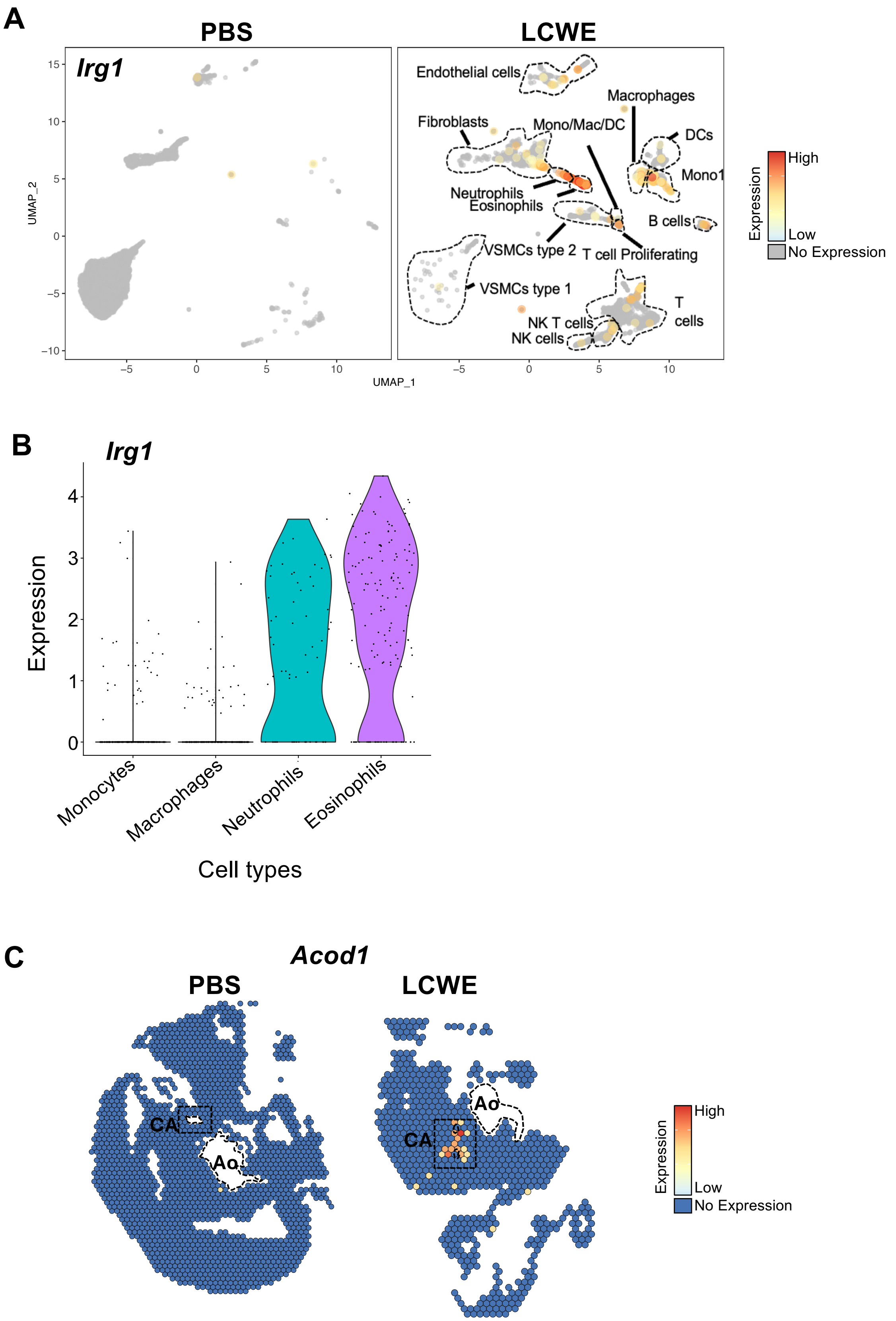

Supplementary Figure 2. *Acod1* is expressed by immune cells infiltrating LCWE-induced cardiovascular lesions.

(A) Uniform Manifold Approximation and Projection (UMAP) visualization of a single-cell RNA-sequencing dataset (GSE178765)[12] generated from the abdominal aorta of PBS (pool of 9 tissues) and LCWE-injected (pool of 7 tissues) mice, and representing a gradient expression of *Acod1 (*also known as *Irg1)* in the different identified cell subsets. Grey-yellow-red gradient: min-max-normalization of CP10K expression. (**B**) Violin plot showing log-transformed normalized *Acod1* (*Irg1*) gene expression in monocytes, macrophages, neutrophils, and eosinophils infiltrating the abdominal aorta dilations of LCWE-injected mice at 2 weeks post-LCWE injection (GSE178765)[12]. (**C**) Analysis of *Acod1* expression in a published spatial transcriptomic dataset (10x Visium; GSE178799[12]) generated from heart tissues of PBS and LCWE-injected mice collected at 2 weeks post-injection. Expression of *Acod1* is limited to the inflamed coronary artery of the heart tissue section from the LCWE-injected mouse. Dark blue spots; no expression of *Acod1*. Light blue-yellow-red gradient: min-max normalization of counts per 10 000 reads expression. CA indicates coronary artery; Ao; aorta.
